# Association between Periodontal Status and Prognosis in Men with Prostatitis: A Cross-Sectional Clinical Study

**DOI:** 10.1055/s-0045-1812498

**Published:** 2025-12-18

**Authors:** Javier Flores-Fraile, Pablo Ortíz de Urbina Comerón, Pedro Molinero-Mourelle, Luca Fiorillo, Cosimo Galletti, Andrea Roccuzzo, María Fernanda Lorenzo Gómez

**Affiliations:** 1 Department of Surgery, Faculty of Medicine and Dentistry, University of Salamanca, Salamanca, Spain; 2 Department of Reconstructive Dentistry and Gerodontology, School of Dental Medicine, University of Bern, Bern, Switzerland; 3 Department of Conservative Dentistry and Orofacial Prosthodontics, Faculty of Dentistry, Complutense University of Madrid, Madrid, Spain; 4 Department of Dental Research Cell, Dr. D. Y. Patil Dental College & Hospital, Dr. D. Y. Patil Vidyapeeth (Deemed to be University), Pimpri, Pune, Maharashtra, India; 5 School of Medicine and Surgery, University of Enna, Enna, Italy; 6 Shanghai Key Laboratory of Stomatology, National Center of Stomatology, National Clinical Research Center for Oral Diseases, College of Stomatology, Shanghai Jiao Tong University, Shanghai, People's Republic of China; 7 Shanghai Perio-Implant Innovation Center, Institute for Integrated Oral, Craniofacial and Sensory Research, Ninth People's Hospital, Shanghai Jiao Tong University School of Medicine, Shanghai, People's Republic of China; 8 Department of Periodontology, School of Dental Medicine, University of Bern, Bern, Switzerland; 9 Biomedical Research Institute of Salamanca (IBSAL), Salamanca, Spain; 10 University Hospital of Salamanca, Salamanca, Spain

**Keywords:** prostatitis, periodontal disease, bleeding index, PSA, oral-systemic health

## Abstract

**Objective:**

Prostatitis is the most prevalent urological condition in young adult men and may progress to prostate cancer, representing a significant public health concern. A high prevalence of inflammatory prostate disorders exists without a clearly identifiable cause. This article aimed to investigate the potential association between periodontal status and prognosis in men diagnosed with prostatitis and to evaluate whether oral health influences urological treatment outcomes.

**Materials and Methods:**

A cross-sectional clinical study was conducted on 172 adult patients with prostatitis who provided written informed consent. Participants were assigned to two groups based on bleeding index (BI): Group A (GA, *n* = 84), BI >7%, and Group B (GB, *n* = 88), BI ≤7%. All subjects underwent comprehensive periodontal assessment and nonsurgical periodontal therapy, with follow-up at 30 and 120 days.

**Results:**

At baseline, GA showed significantly greater probing depths on proximal surfaces and higher BI values than GB ( *p* < 0.01). Posttreatment, BI improved significantly in both groups, although GA maintained higher residual bleeding ( *p* = 0.002). Mean posttreatment prostate-specific antigen (PSA) levels did not differ significantly between groups; however, PSA decreases occurred more frequently in GB (61.5%) than in GA (28.6%, *p* = 0.039), while PSA increases were more common in GA (57.1% vs. 15.4%, *p* = 0.005). Overall, 29.4% of patients exhibited lower PSA levels after periodontal therapy.

**Conclusion:**

While no direct association was found between periodontal status and absolute posttreatment PSA values, healthier periodontal conditions were linked to more favorable PSA trends. These findings suggest that periodontal health may contribute to PSA stability in men with prostatitis. Larger, longitudinal studies are warranted to clarify potential mechanistic links between oral and prostatic inflammation.

## Introduction

In recent years, several published articles have supported the link between periodontal disease and other conditions, including neurodegenerative diseases (Alzheimer's disease), 1 metabolic disorders (type II diabetes), systemic disorders (rheumatoid arthritis, psoriasis, Parkinson's disease), and certain types of cancer (head and neck, lung). These findings have led to the recognition that periodontal disease is not solely an isolated oral condition, as it has systemic effects. 2 3 4 5 6 7

Some pathogenic bacteria present in the oral cavity, such as *Fusobacterium nucleatum* , *Tannerella forsythia* , *Treponema denticola* , *Porphyromonas gingivalis* , and *Aggregatibacter actinomycetemcomitans* , have been directly and positively associated with pancreatic, gastrointestinal, and oral cancers. 8 These bacteria are capable of regulating proinflammatory cytokines and chemokines (including interleukin [IL]-1β, IL-6, tumor necrosis factor [TNF]-α, and C-reactive protein), which can damage both the oral and systemic immune systems, potentially triggering oncogenic responses.

Given the high prevalence of inflammatory prostate pathology of unknown origin and the beneficial effects of periodontal treatments, the hypothesis arises that periodontal disease could be a contributing factor.

Prostatitis is the most common urological disease in young adults, with an estimated lifetime prevalence of 9 to 14%. 9 The pathophysiological mechanisms and epidemiological links between prostatitis and other diseases are not well defined. However, three mechanisms attempt to explain a possible interrelation between periodontitis and prostatitis. One direct method, supported by Estemalik in 2017, proposes bacterial migration via the bloodstream to the prostate. Estemalik observed that 17 out of 24 patients had at least one oral pathogen present in their prostatic secretions. 10

The discovery of prostate-specific antigen (PSA) marked a turning point in the diagnosis of prostate cancer (PC). Although the standard PSA values fall within the range of 0 to 4 ng/mL, 11 a PSA level above 4 is not necessarily indicative of PC, as it can be elevated due to prostatitis, benign prostatic hyperplasia (BPH), or after prostatic manipulation. It can also be lowered by the intake of insulin or aspirin. 12

PSA is thus a continuous parameter, with a higher probability of prostate cancer associated with higher values. Many men can develop prostate cancer even while maintaining low PSA serum levels.

In 1980, Papsidero and his collaborators pioneered the quantification of PSA levels in human blood, but it was not until the 1990s that PSA became routinely used as a diagnostic tool. 13 14 In the United States, a PSA cutoff value of 2.5 ng/mL was established, although prostate biopsy was typically indicated when PSA levels exceeded 4 ng/mL. 7

In 2012, 1.1 million cases of prostate cancer were diagnosed, and 307,000 deaths were attributed to this condition. These overwhelming statistics suggest that one in seven men will develop prostate cancer in their lifetime, making it one of the most prevalent diseases today. The probability of a man developing this cancer is 17%, while the likelihood of death from the disease is 3%. 15

There is evidence that the oral microbiome, in cases of advanced periodontal disease, can spread beyond the oral cavity through local inflammation, bone destruction, and tooth loss. 16 17 Recent population-based research also supports this connection, showing that recurrent periodontitis is significantly associated with an increased incidence of BPH. 18

## Materials and Methods

A cross-sectional clinical study is conducted to investigate the association between prognosis in men diagnosed with prostatitis and their periodontal status. The study takes place at the Dental Clinic of the University of Salamanca, which is equipped with 45 dental chairs, allowing for the simultaneous treatment of multiple patients and ensuring an adequate clinical environment for data collection.

The Urology Department of the Clinical University Hospital of Salamanca provides a registry of all patients diagnosed with prostatitis between 2012 and 2022, yielding a total of 249 potential patients. After a selection process and obtaining informed consent, 172 patients agreed to participate in the study.

They are thoroughly informed about the research objectives and the procedures involved, ensuring their full understanding and voluntary participation. All participants undergo a prior blood analysis, which includes measurement of PSA levels.

The sample is divided into two groups:

- **Group A (GA):** Patients exhibiting evident periodontal disease (tooth mobility, bleeding, or tooth loss) with a bleeding index >7%.
- **Group B (GB):** Patients with optimal periodontal status (no bleeding, no probing, no tooth loss) with a bleeding index <7%.

The inclusion criteria for the study are as follows: age > 18 years, diagnosis of prostatitis, and willingness to participate in the study (signed informed consent).

All patients underwent a dental and periodontal clinical history and examination. The study was conducted in four phases:

- ***Day 0*:** Oral examinations are conducted, and periodontal charts, Community Periodontal Index (CPI), and bleeding indices are filled out.
- ***Day 1*:** Nonsurgical periodontal treatment is performed.
- ***Day 30*:** Posttreatment periodontal examination is conducted.
- ***Day 120*:** Posttreatment analytical evaluation is performed.

For the study, a World Health Organization (WHO)-type periodontal probe is used, specifically designed with a rounded tip (ball), a diameter of 0.5 mm, and a black marking between 3.5 and 5.5 mm.

To evaluate the periodontal data, the Community Periodontal Index (CPI) is employed. The mouth was divided into sextants limited by the canines, excluding third molars. A sextant is considered valid if it contains at least two functional teeth; if not, it is deemed invalid.

The index teeth examined are: 16–26, 36–46, 21, and 41. For each sextant, the worst score among these index teeth is recorded. If any of the specified teeth are absent, all surfaces of the relevant sextant are examined, and the highest value found is noted.

The coding system used in the study is as follows:

- **0:** Healthy: No bleeding, calculus, or periodontal pockets when the probe is inserted.
- **1:** Hemorrhage: Bleeding occurs upon gentle probing, with no calculus or periodontal pockets present.
- **2:** Calculus: Presence of bleeding and supragingival and subgingival calculus. Periodontal pockets are present; however, the dark zone of the probe is completely visible.
- **3:** Pocket of 3.5 to 5.5 mm: Presence of bleeding and supragingival and subgingival calculus. Periodontal pockets are present; however, the dark zone of the probe is partially visible.
- **4:** Pockets of >5.5 mm: Presence of bleeding and supragingival and subgingival calculus. Periodontal pockets are present; the dark zone of the probe is not visible.

The above codes indicate specific treatment needs for each sextant:

- **NT 0:** No treatment needed (code 0).
- **NT 1:** Needs instruction in oral hygiene (IOH) (code 1).
- **NT 2:** Removal of calculus and/or overhanging restorations (codes 2 and 3): IOH + scaling and root planing (SRP).
- **NT 3:** Requires complex treatment (code 4): IOH + SRP + periodontal surgery.

### Statistical Analysis

For the statistical analysis, both qualitative and quantitative variables were analyzed using the NCSS277/GESS2006 statistical software. Baseline characteristics of the study population (age, comorbidities, and periodontal status) were compared between groups to ensure consistency at the start of the study. Quantitative variables were assessed using Student's *t* -test or Mann–Whitney *U* -test, as appropriate, and categorical variables were compared using the chi-square test. No significant differences were observed, confirming the homogeneity of the groups at baseline.

## Results

### General Characteristics of the Sample

Of the 249 patients registered with a diagnosis of prostatitis, 172 met the inclusion criteria and participated in the study. GA (>7%) included 84 patients (48.8%), while GB (bleeding index ≤7%) included 88 patients (51.2%). The mean age of the total sample was 59.13 ± 8.4 years.

### Baseline Probing Depth (Day 0)

At baseline periodontal examination, significantly greater probing depths were observed in GA on mesial and distal surfaces ( Table 1 ). No significant differences were found for vestibular or lingual surfaces ( Fig. 1 ).

**Table 1:** Baseline probing depth by surface

Surface	GA (mean ± SD, mm)	GB (mean ± SD, mm)	*p* -Value
**Mesial**	2.32 ± 0.70	2.03 ± 1.05	**0.003**
**Distal**	2.42 ± 0.66	2.00 ± 0.89	**0.001**
**Vestibular**	1.74 ± 0.62	1.80 ± 1.09	0.429
**Lingual**	1.90 ± 0.65	1.87 ± 1.11	0.226

Abbreviations: GA, Group A; GB, Group B; SD, standard deviation.

Note: Numbers in bold denote significance.

**Fig. 1:**
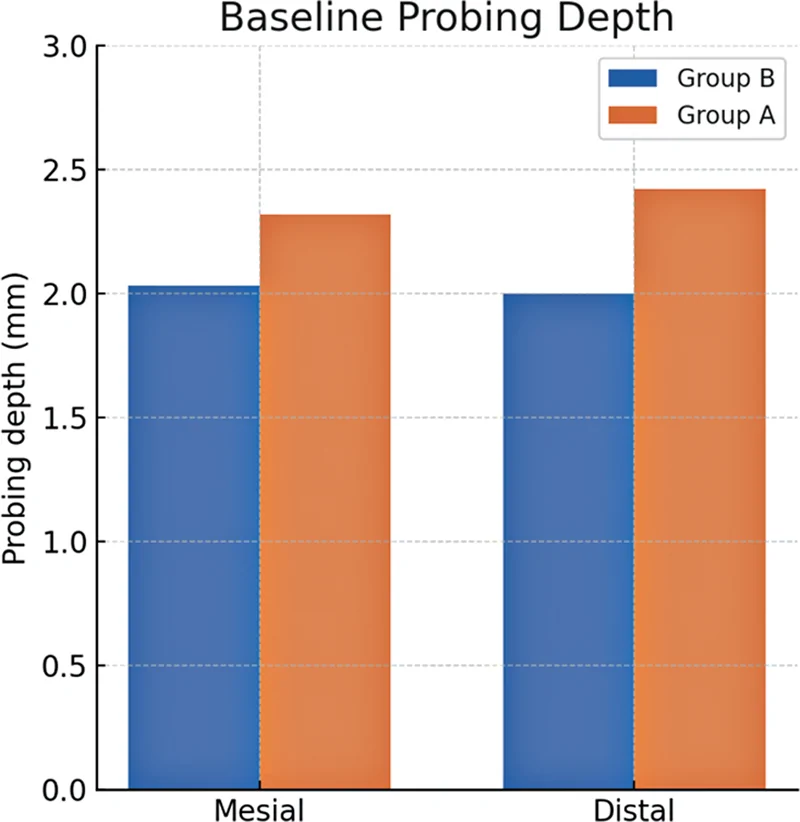
Bar chart showing baseline mesial and distal probing depths in both groups.

### Bleeding Index Evolution

The initial bleeding index was significantly higher in GA (15.16 ± 7.23%) than in GB (2.70 ± 2.30%; *p* < 0.001; Table 2 ). Following periodontal treatment, both groups exhibited a marked reduction, though GA maintained higher residual values ( Fig. 2 ).

**Table 2:** Bleeding index progression (%)

Time point	GA (mean ± SD)	GB (mean ± SD)	*p* -Value
Baseline	15.16 ± 7.23	2.70 ± 2.30	**<0.001**
Posttreatment	3.13 ± 3.83	0.82 ± 1.41	**0.002**

Abbreviations: GA, Group A; GB, Group B; SD, standard deviation.

Note: Numbers in bold denote significance.

**Fig. 2:**
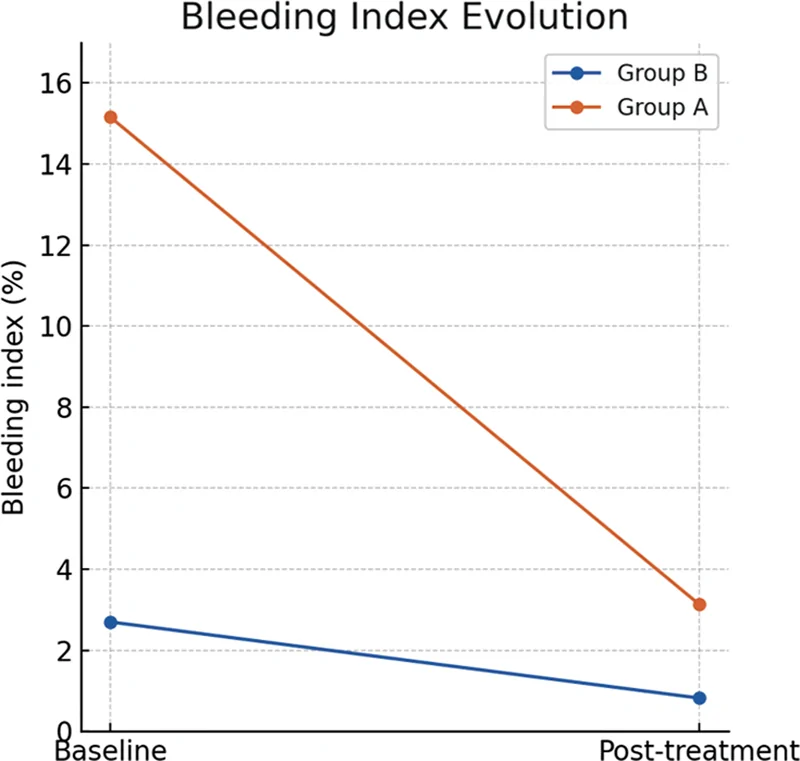
Line graph depicting bleeding index reduction from baseline to posttreatment.

### Posttreatment PSA Levels

Mean post-treatment PSA did not differ significantly between groups (GA: 2.19 ± 4.07 ng/mL; GB: 1.71 ± 2.19 ng/mL; *p* = 0.622; Table 3 ). However, the distribution of individual PSA changes differed ( Fig. 3 ):

**Table 3:** Changes in PSA posttreatment

PSA change	GA, *n* (%)	GB, *n* (%)	*p* -Value
Decrease	24 (28.6)	54 (61.5)	**0.039**
No change	12 (14.3)	20 (23.1)	0.711
Increase	48 (57.1)	14 (15.4)	**0.0048**

Abbreviations: GA, Group A; GB, Group B; PSA, prostate-specific antigen.

Note: Numbers in bold denote significance.

**Fig. 3:**
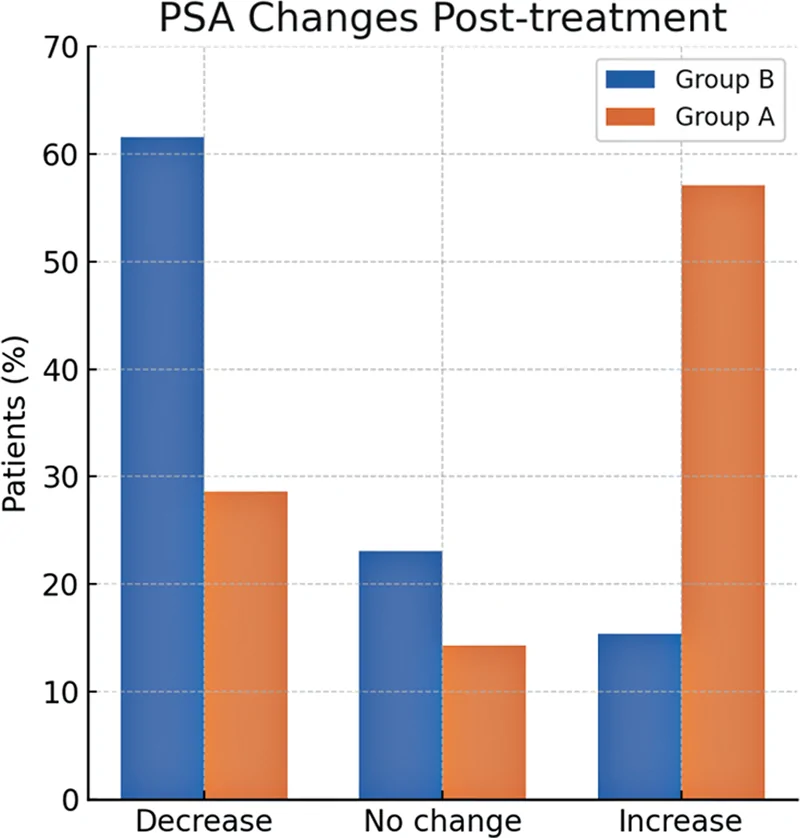
Grouped bar chart illustrating the distribution of PSA changes posttreatment.

- PSA increase: more frequent in GA (57.1%) than in GB (15.4%) ( *p* = 0.005).
- PSA decrease: more frequent in GB (61.5%) than in GA (28.6%) ( *p* = 0.039).

## Discussion

Periodontal disease has increasingly been recognized as not merely a local oral condition but as a systemic health concern. Evidence from epidemiological and mechanistic studies has linked periodontitis to numerous chronic diseases, including cardiovascular disorders, diabetes, neurodegenerative conditions, and several types of cancer. 19 20 21 These associations are thought to arise through multiple mechanisms, including local dysbiosis, chronic systemic inflammation, immune evasion, and direct (epi)genetic effects of periodontal pathogens and their toxins. 19 Such findings highlight the broader significance of oral health in the prevention and management of systemic diseases.

Specifically concerning prostatic health, recent studies suggest a significant association between periodontal disease and prostatic inflammation, BPH, and potentially prostate cancer. Ortíz de Urbina Comerón et al performed a systematic review and meta-analysis demonstrating that periodontal disease is significantly associated with increased incidence of prostatic inflammation, supporting a role for chronic oral infections in urological pathology. 20 22 Similarly, Wu et al reported that periodontitis is positively correlated with the risk of developing BPH, implying that systemic inflammation originating from oral infections may contribute to prostate tissue proliferation and remodeling. 23

Mechanistic insights reinforce these epidemiological findings. Wang et al identified that *P. gingivalis* , a key periodontal pathogen, can exacerbate BPH via the IL-6/IL-6R signaling pathway. 24 This provides a plausible molecular mechanism linking oral infections to distant organ pathology, suggesting that chronic oral inflammation may directly influence prostatic tissue through systemic inflammatory mediators. Such pathways mirror those implicated in other chronic inflammatory conditions, reinforcing the concept of periodontitis as a systemic disease with far-reaching effects.

Evidence regarding the relationship between periodontal disease and prostate cancer remains more heterogeneous but still noteworthy. Several meta-analyses and cohort studies suggest that men with periodontitis may have an elevated risk of developing prostate cancer. 6 21 However, some systematic reviews highlight variability and inconclusive results, likely due to differences in study populations, diagnostic criteria for periodontitis, and methodological heterogeneity. 22 Mechanistically, chronic systemic inflammation and persistent oral dysbiosis may promote carcinogenic processes via oxidative stress, DNA damage, and immune modulation in prostatic tissue, paralleling mechanisms observed in other cancers associated with periodontal disease. 19 22

The clinical implications of these findings are substantial. Maintenance of oral hygiene and effective treatment of periodontitis could reduce systemic inflammatory burden and thereby mitigate risks of prostatic inflammation, BPH, and potentially prostate cancer. These observations suggest a potential benefit of integrated care involving dental and urological management, particularly for patients at high risk for chronic prostatic conditions. 20 21 24

Considering the high prevalence of periodontitis and the global burden of prostate-related disorders, these findings underscore the importance of preventive oral healthcare as a potential strategy to improve systemic outcomes. Future longitudinal and mechanistic studies are warranted to clarify the exact pathways connecting oral pathogens and prostatic pathology, and to evaluate the therapeutic impact of periodontal interventions on prostate-related health outcomes.

Recent research further supports this connection. A 2025 study explored the intersection of prostatitis and periodontitis, reinforcing the systemic nature of these conditions and suggesting that oral inflammation may directly exacerbate prostatic pathology. 25 Moreover, updated reports from. Recent evidence underscores the importance of integrating dental and urological perspectives when evaluating chronic prostatic conditions. Incorporating these findings reinforces the idea that periodontal disease management may represent a novel preventive strategy against prostate-related disorders. 26

## Conclusion

In this cross-sectional clinical study, no direct association was observed between periodontal status and absolute post-treatment PSA values in men with prostatitis. However, patients with a high bleeding index (GA) showed greater baseline probing depths, higher initial bleeding indices, and a higher frequency of PSA increases after periodontal therapy. Conversely, patients with better periodontal health (GB) more frequently exhibited PSA reductions. Although periodontal treatment significantly improved bleeding indices in both groups, residual bleeding remained higher in GA.

These results indicate that periodontal status may influence PSA dynamics, with healthier periodontal conditions being associated with more favorable PSA trends. The findings support the potential role of periodontal therapy as an adjunctive approach to reduce systemic inflammatory burden in men with prostatitis. Future longitudinal studies with larger cohorts are warranted to confirm these associations and to further elucidate the underlying biological mechanisms linking periodontal disease and prostatic inflammation.
